# A New Therapeutic Approach With Rose Stem-Cell-Derived Exosomes and Non-Thermal Microneedling for the Treatment of Facial Pigmentation

**DOI:** 10.1093/asjof/ojae060

**Published:** 2024-08-09

**Authors:** Elina Theodorakopoulou, Shino Bay Aguilera, Diane Irvine Duncan

## Abstract

**Background:**

Facial dyspigmentation is a challenging concern which cannot easily be corrected. Although the application of topical exosomes has shown some efficacy, there is still scarce data addressing the role of plant-derived exosomes for skin hyperpigmentation.

**Objectives:**

This study using rose stem-cell-derived exosomes (RSCE) was performed as a proof-of-concept case series to evaluate the efficacy and safety of microneedling and topical RSCE, for the reduction of pigmentation and photoaging in adult volunteers.

**Methods:**

Twelve female volunteers were recruited, with a mean age of 46.64 years and a moderate-to-severe facial pigmentation, due to solar lentigines, melasma, postinflammatory hyperpigmentation, and periorbital hyperpigmentation. Three treatments were performed at 3 weeks intervals. These consisted of the topical application of RSCE with microneedling and a 20 min LED light with an RSCE-infused mask. A 3D facial analyzer was used to quantify improvement in superficial, deep pigmentation, skin redness, and wrinkles at baseline, Weeks 3, 6, and 12. Global Aesthetic Improvement Scale (GAIS), Dermatology Life and Quality Index (DLQI), and Melasma Quality of Life Scale (MELASQoL) scores were noted at the same time points.

**Results:**

GAIS scores improved by at least 1 scale point. Superficial pigmentation and spots decreased by 12.95% and deep pigmentation improved by 15.9%, by Week 12. Skin redness was reduced by 7.34% at the same time point. The measured wrinkle reduction was 6.34%. DLQI scores were reduced by 10 points, and MELASQoL scores had a mean reduction of 30 points at Week 12.

**Conclusions:**

Improvement of facial pigmentation is possible when combining nonthermal microneedling and the use of topical RSCE.

**Level of Evidence: 4:**

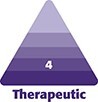

Skin pigmentation is a major skin concern that affects all skin types and is associated with a high perceived stigma.^1,2^ The spectrum of conditions defined as pigmentary disorders include solar lentigines (age/sun spots), on chronically sun-exposed skin (face, top of hands), postinflammatory hyperpigmentation (PIH) that results in increased pigment at the site of inflammation (insect bite, injury, acne scars, and others), and the very common melasma characterized by tan, brown patches, typically affecting the face (forehead, cheeks, upper lip). There are multiple factors that can contribute to the various pigmentary disorders that exist, including genetic, hormonal, inflammatory (inflammation, autoimmune disease), environmental (ultraviolet [UV] radiation) factors, as well as various medications.^3^ At the same time, there may be multiple treatments to help with pigmentary skin lesions but there is no permanent solution or cure.

Pathomechanism of human pigmentation system is complex and not necessarily confined to melanocytes.^4^ There is a multiplex interplay between cellular communications and signaling pathways, including autocrine and paracrine mechanisms, on epidermal/dermal level that, once dysregulated, leads to pigmentary disorders.^2^ UV exposure is the main environmental trigger that influences skin pigmentation, and 1 significant pathway that is activated is through alpha-melanocyte-stimulating hormone (α-MSH), secreted by keratinocytes and melanocytes.^5^ Further adding to the complexity of pigmentary disorders, melanin synthesis is triggered by alterations in the function of dermal fibroblasts and the homeostasis of the extracellular matrix. To add to the previous, in vitro and 3D experimental studies confirm the positive relationship between aged/photoaged dermal fibroblast and increased melanogenesis, leading to pigmentary lesions.^2,4,5^

Exosome-derived products for the skin are currently used as dermocosmetics, for topical application, and they seem to be promising for both skin aging and pigmentary lesions. Exosomes are nanosized (50-150 nm) membrane vesicles that deliver therapeutic messages to the target tissue/cell and therefore consist of promising therapeutic platforms for skin repair and rejuvenation.^6^ Preclinical studies on mesenchymal stem-cell-derived exosomes (MSC-Exos) have displayed the efficacy of MSC-Exos on reversing the skin aging phenotype.^7,8^ Lo Cicero et al’s experimental skin model demonstrated the communication of keratinocytes and melanocytes through exosomes that have the ability to modulate pigmentation.^9^ Recently, Cho et al demonstrated, in vitro, the antipigmentary properties of adipose tissue stem-cell-derived exosomes (ASCE), despite the presence of α-MSH.^10^ Further added, clinical split-face studies on ASCE displayed significant improvement of pigmentary lesions, improvement of acne scars post-CO_2_ laser, and cutaneous aging, after microneedling, in the study group using either topical application of ASCE compared with the control group.^11,12^ Similar findings on the improvement of skin quality and tone were reported by Chernoff when he investigated the topical application of or human placental mesenchymal stem-cell-derived exosomes (hPMSC-exos) on human skin, with microneedling.^13^

In a recent in vitro study, Won et al have presented a novel exosome-like particle derived from rose stem cells.^14^ The authors demonstrated that rose stem-cell-derived exosomes (RSCE) effectively induced the proliferation of human fibroblast and the subsequent production of collagen and elastin. When uptaken by melanocytes, RSCE are noted to reduce melanin synthesis. Moreover, when RSCE targeted macrophages, they reduce the levels of interleukin-6 (IL-6), exhibiting as such antiinflammatory properties.

To better amplify the results of topically applied exosomes on the skin, devices that are utilized to form micropores in the skin are ideal to allow these tiny nanoparticles to enter the deeper layers of the skin. Microneedling is a safe and effective method to mechanically induce skin repair and remodeling, improving skin texture, the appearance of scars, and striae, and it has been extensively used in all skin types, with good results also on darker skin phenotypes.^15^ A large volume of published studies has charted the facilitated permeability of therapeutic molecules (small molecules, nanosized medicines, exosomes, live cells, and others) through the skin, by creating reversible microchannels in the skin, with microneedling.^16-18^ In a recent work, Park et al have supported the coworking effect of topical ASCE and microneedling for skin rhytids.^12^

Based on the abovementioned promising results, the authors hypothesized that the combination of microneedling, with the topical application of RSCE, may potentially improve skin aging and pigmentary lesions. The objective of this study was to evaluate the antipigmentary, antiaging effects of RSCE with microneedling.

## METHODS

### Study Design

This open-label, uncontrolled study was conducted between September 2023 and December 2023, to evaluate the efficacy and safety of topical application of RSCE with microneedling, for skin hyperpigmentation and photoaging. Primary endpoint was to evaluate whether the combination treatment of RSCE with microneedling improves skin pigmentation, based on clinical and noninvasive instrumental grading. An improvement of the severity of skin pigmentation by at least 1 point in the clinical grading systems and a significant improvement in the instrumental grading systems were considered successful results. Secondary outcome measures included the subsequent improvement of skin photoaging and inflammation, as measured by the severity of wrinkles and erythema, while tolerability and safety of treatment were also assessed.

### Setting

Twelve otherwise healthy patients were recruited by Dr Elina Theodorakopoulou, good clinical practice–trained, Dermatologist in Athens, Greece, from 1 study center based in Athens, Greece.

### Participants

This study was conducted in accordance with the principles of the Declaration of Helsinki, and all participants provided written informed consent prior to enrollment. This study adheres to ethical guidelines. The inclusion criteria consisted of healthy patients, over the age of 18 years, with moderate-to-severe photoaging and hyperpigmentation. Patients diagnosed with either solar lentigines, melasma, PIH, and/or periorbital hyperpigmentation, for at least 6 months, were eligible to take part in the study. Severity of skin quality and pigmentation were assessed using the 5-point photonumeric Scientific Assessment Scale of Skin Quality (SASSQ) for aged skin (Table 1), by Eiben-Nielson and Kerscher,^19^ whereas melasma lesions were scored using the 4-point Melasma severity scale (MSS) (Table 2), described by Taylor et al^20^ In total, 5 patients with melasma, 3 patients with lentigines, and 4 patients with PIH were recruited to the study. Other inclusion criteria consisted of patients of childbearing potential, who used an acceptable method of contraception throughout the study and patients willing to withhold all facial treatments during the study, including botulinum toxin, injectable fillers, peels, facials, and laser treatments, including facial laser hair removal.

**Table 1.** The 5-Point Photonumeric Scientific Assessment Scale of Skin Quality for Aged Skin

Rating	Description
A.	
0	No visible pigmentation
1	Mild pigmentation, lentigines
2	Moderate pigmentation, lentigines
3	Severe pigmentation, lentigines
4	Very severe pigmentation, lentigines
B.	
0	No visible wrinkles
1	Mild wrinkles
2	Moderate wrinkles
3	Severe wrinkles
4	Very severe wrinkles
C.	
0	No visible erythema
1	Mild erythema
2	Moderate erythema
3	Severe erythema
4	Very severe erythema

**Table 2.** The 4-Point Melasma Severity Scale for Melasma Lesions

Rating	Description
0	Cleared: color of melasma lesions approximately equivalent to surrounding normal skin or with minimal residual hyperpigmentation
1	Mild: color slightly darker than the surrounding normal skin
2	Moderate: color moderately darker than the surrounding normal skin
3	Severe: color markedly darker than the surrounding normal skin

Exclusion criteria included pregnancy, autoimmune conditions, malignancy, history of keloids, active skin disease, contagious skin disease, tattooing on the face, and treatments for hyperpigmentation or skin rejuvenation, including topical creams (Kligman's formula, hydroquinone, vitamin C, α- and β-hydroxy acids, and other bleaching compounds), lasers, radiofrequency microneedling, peelings, injectable fillers, skin-brightening mesotherapy solutions, toxins, surgical lifting, and oral intake of vitamin C, tranexamic acid, retinoids, or phototoxic/photoallergic oral medications, for the past 6 months, before study enrollment.

### Treatment Protocol

Study visits were conducted at enrollment, baseline, Weeks 3, 6, and 12. Each participant received 3 treatments, at 3 weeks intervals, whereas the follow-up information was obtained at Week 12, 6 weeks after the last treatment. Before each treatment, make-up was thoroughly removed with cleansing micellar water, and the face was then cleansed with mild soap and disinfected with hypochlorous acid. Local anesthetic cream was not applied in the skin, to avoid changes in the vasculature. The topical blend of RSCE was then prepared, using the 2 vials from the ASCE^plus^ Dermal Signal Kit (ExoCoBio, Korea). The Vial 1 which contains 20 mg lyophilized powder of RSCE (5 × 10^9^ particles) was mixed with Vial 2, the 5 mL diluent solution, containing aminoacids, growth factors, noncross-linked, hyaluronic acid, vitamin C, and retinol. The product is available in Europe and registered at the Cosmetic Product Notification Portal. Half of the final blend (2.5 mL) was then applied to the patient's face, and a microneedling treatment session was followed, all over the face, at a depth of 1 mm, whereas for periorbital, perioral, and nasal skin at a 0.2 mm depth (Dermapen 4, Australia). A total of 2 passes in each area, in vertical, horizontal, and oblique directions (density) were performed. Another 2.5 mL of the RSCE blend was applied topically 5 min later and gently massaged on the treated skin. An RSCE-infused mask (ASCE^plus^ soothing gel mask, ExoCoBio, Korea) was applied to the treated skin, and patients’ face was placed under red light (635 nm wavelength) for 20 min (LED PDT photodynamic therapy, Greece). Posttreatment aftercare advice included the use of a broad spectrum, high sun protection factor (>30) sunscreen, with iron oxide, and a bland moisturizer. In addition, patients were advised to follow a low histamine diet and avoid sun exposure, tanning beds, and face or UV nail driers, for the duration of the study. They were also advised to refrain from any antiaging, depigmenting topical agents, and the use of any new cosmeceutical products, apart from the ones assigned for the study.

### Clinical Evaluation

Clinical photography of each participant was documented at each visit, at 5 different positions; lateral views (left, L and right, R), frontal view, oblique R + L views (iPAD Pro, 6th Generation, Apple, Cupertino, California). The Global Aesthetic Improvement Scale (GAIS)^21^ was employed to evaluate the aesthetic improvements clinically and subjectively (Table 3). GAIS was rated independently by an independent evaluator and the patient at 3 different time points. Baseline clinical photographs of study patients were compared with their clinical photographs taken at Weeks 3, 6, and 12. To clinically assess the improvement of skin quality and pigmentation, the SASSQ and MSS were completed at the baseline visit and Week 12. Aesthetic improvement on GAIS was defined as “improved,” “much improved,” or “very much improved.” Adverse events, including erythema, edema, bruising, scarring, pain/tenderness, pruritus, infection, inflammation, pigmentation, nodules, and/or atrophic/hypertrophic lesions, were documented at each time point.

**Table 3.** Global Aesthetic Improvement Scale Assessment for the Evaluation of Treatment Response

Rating		Description
1	Very much improved	Optimal cosmetic result for the patient
2	Much improved	Marked improvement in appearance from the initial condition, but not completely optimal for the patient
3	Improved	Obvious improvement in appearance from initial condition, but a retreatment is indicated
4	No change	The appearance is essentially the same as the original condition
5	Worse	The appearance is worse than the original condition

### Noninvasive Instrumental Evaluation

The objective measurement of improvement of dyspigmented lesions and facial wrinkles was performed using a standardized skin analysis imaging system, using a 3 spectral imaging technology (3D Magic Mirror Facial skin analyzer, Sincoheren S&T Development Co., Ltd, Beijing, China). The device uses different types of light to detect and evaluate different skin characteristics. In standard light, wrinkles and pore size are identified. In UV light mode, the device quantifies hyperpigmented spots and porphyrin. Finally, in polarized (PL) RGB LED (red–green–blue, light emitting diode) light, brown, red areas, and vascular lesions are evaluated (red/brown surface analysis). Four parameters were assessed in each visit: percentage of superficial pigmentation (sp), percentage of deep pigmentation (dp), percentage of skin redness (sr), and percentage of nonwrinkled skin (nw). The ambient temperature (°C) and relative humidity (RH) during the measurements were 21 ± 1 °C and 50% ± 10% RH, respectively. Before the assessment, patients were asked to wash their faces with water and to acclimatize with the temperature of the room for 20 min.

### Patient Satisfaction and Self-Evaluation

The patient's subjective appreciation of self-improvement posttreatment was assessed using GAIS at Weeks 3, 6, and 12. In addition, patients were asked to document changes in health-related quality of life (HRQoL) pre- and posttreatment, through completing the Dermatology Life and Quality Index (DLQI)^22^ and the Melasma Quality of Life Scale (MELASQoL).^23-25^ DLQI and MELASQoL were completed by the patients at baseline and Week 12. DLQI is a 10-item questionnaire, with questions referring to symptoms, feelings, daily activities, leisure, work, personal relationships, and treatment. Each question is answered on a Likert scale of 0 to 3: “not at all,” “a little,” “a lot,” or “very much.” The scores of each answered question are summed, giving a range from 0 (no impairment of life quality) to 30 (maximum impairment). All questions relate “to last week.” The MELASQoL consists of 10 questions, each one scored on a Likert scale of 1 to 7 in which 1 signifies not bothered at all and 7 signifies bothered all the time. MELASQoL scores range from 10 to 70, with higher scores indicating worse HRQoL.

### Statistical Analysis

IBM-SPSS Statistics 26 was used for statistical analysis. All data were assessed for normality with Shapiro–Wilk test. As the majority of parameters were nonparametric, the Friedman's 2-way analysis of variance by ranks tests was used for repeated measures of >2 paired groups (percentage of sp and dp, percentage of sr, percentage nw, GAIS), whereas the Wilcoxon signed-rank test was employed to compare up to 2 paired groups (SASSQ, MSS, DLQI, and MELASQoL). The Spearman's rank correlation was used to estimate significant correlations between MELASQoL and DLQI. The statistical significance level was established at *P* < .05.

## RESULTS

Patients’ demographics included 12 female volunteers (mean age 46.64 ± 13.05 years), predominantly of Caucasian origin, with Fitzpatrick skin phototypes between II and IV.

### Clinical Evaluation

**Figure 1.**
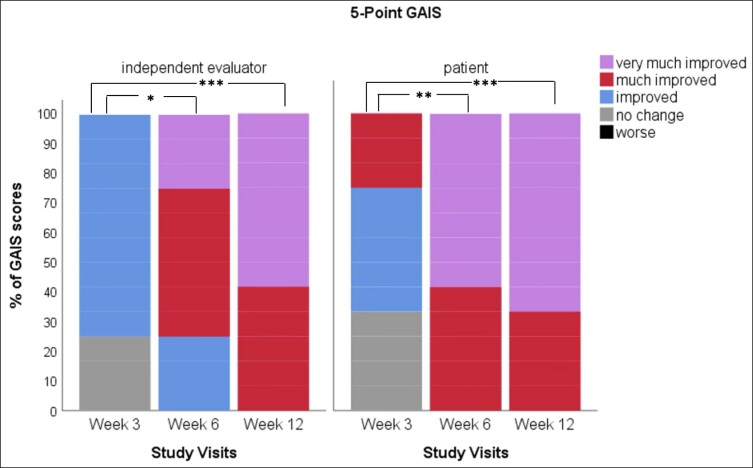
Global Aesthetic Improvement Scale (GAIS) scores. The bar chart shows the significant improvement of aesthetic perceptions in GAIS as recorded by both study patients and an independent evaluator, at different time points. Patients underwent 3 subsequent treatments at baseline, Week 3, and Week 6, with topical application of rose stem-cell-derived exosomes and microneedling. *P < .05, **P < .01, ***P < .001.

There was a significant improvement in GAIS both documented by the patient (Friedman's test, *x*^2^ = 17.526, *P* = .000) and independent evaluator (*x*^2^ = 21.415, *P* = .000). Even after the first treatment, both patient and independent evaluator, at Week 3, documented at least 1 point, in aesthetic improvements on GAIS, with 42% of patients scoring “improved” and 25% of patients “much improved,” whereas the rest 33% observed “no change” from baseline. With respect to the patient-evaluated GAIS scores, after the second treatment, there was a significant improvement on aesthetic appearances compared with first treatment (mean rank = 1.63, adjusted *P* = .007), with 58% of patients rating their condition as “very much improved” and 42% as “much improved.” Similar scores were recorded by the independent evaluator (mean rank = 1.79, adj. *P* = .013). Most notably, on the patient-evaluated GAIS, there was no significant difference in aesthetic improvements between Weeks 6 and 12, as 67% of patients reported “very much improved” compared with 58% in Week 6 (mean rank 1.50, adj. *P* = 1.000). For the independent evaluator, 58% of participants were “very much improved” and the rest “much improved,” while again there was no significant difference in aesthetic improvements between second and third treatments (mean rank = 1.25, adj. *P* = .554). Most importantly, there was no worsening of the skin condition or any relapse, according to GAIS scores (Figure 1).

In line with the previous results, based on the SASSQ and MSS scores, skin hyperpigmentation significantly ameliorated at Week 12, compared with baseline, with a 2-point improvement in severity (from moderate-to-sever pigmentation at baseline to mild or cleared at Week 12; Wilcoxon signed-rank test, *Z*_SASSQ_ = −2.530, adj. *P* = .011; *Z*_MSS_ = −2.121, adj. *P* = .034).

No serious adverse events were recorded. The most common treatment-related adverse effects were mild-to-moderate erythema for <24 h posttreatment (83% of patients), mild edema <24 h posttreatment (67%), pain during the treatment (25%), and mild pruritus < 48 h posttreatment (33% of patients).

### Instrumental Grading

**Figure 2.**
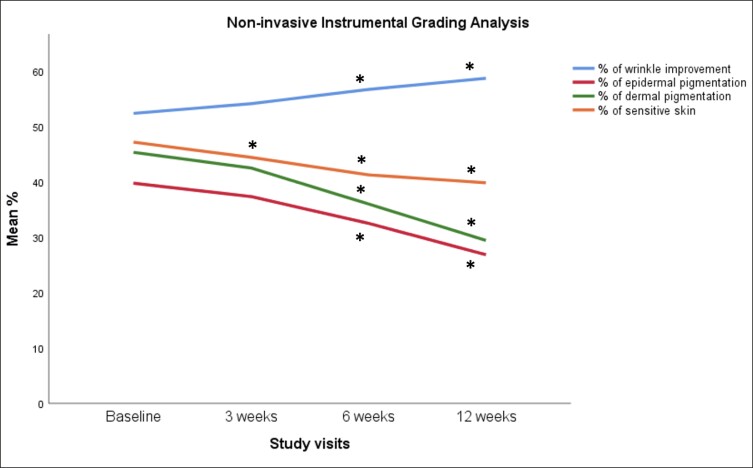
The effect of rose stem-cell-derived exosomes (RSCE) on photodamaged, hyperpigmented skin. The diagram shows the percentage of improvement of ultraviolet-induced pigmentation in the superficial and deep layers of the skin, the percentage of improvement of red inflamed areas of the face, and the percentage of increase of nonwrinkled skin, at the different study visits. Patients underwent 3 subsequent treatments at baseline, Week 3, and Week 6, with topical application of RSCE and microneedling. *P ≤ .001.

**Figure 3.**
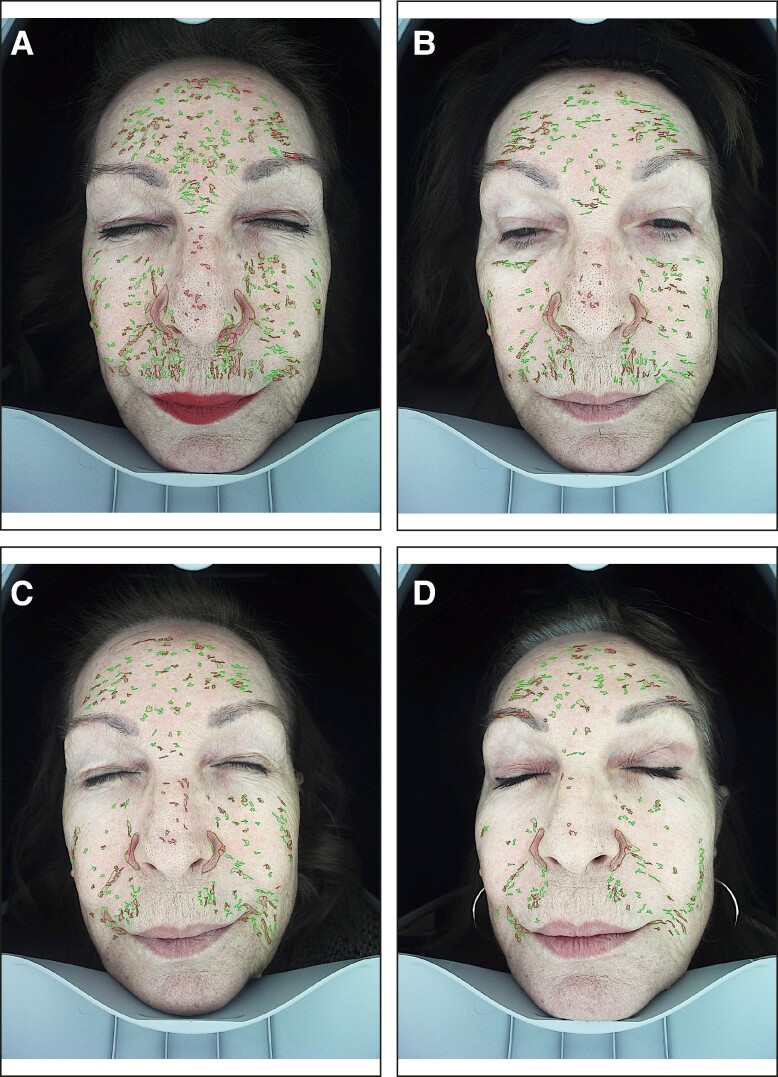
Imaging analysis of long-term ultraviolet-induced pigmentation (age spots), in a 60-year-old female patient. (A) Facial pigmentation before treatment. (B-D) Improvement of pigmentation at Weeks 3, 6, and 12, respectively.

**Figure 4.**
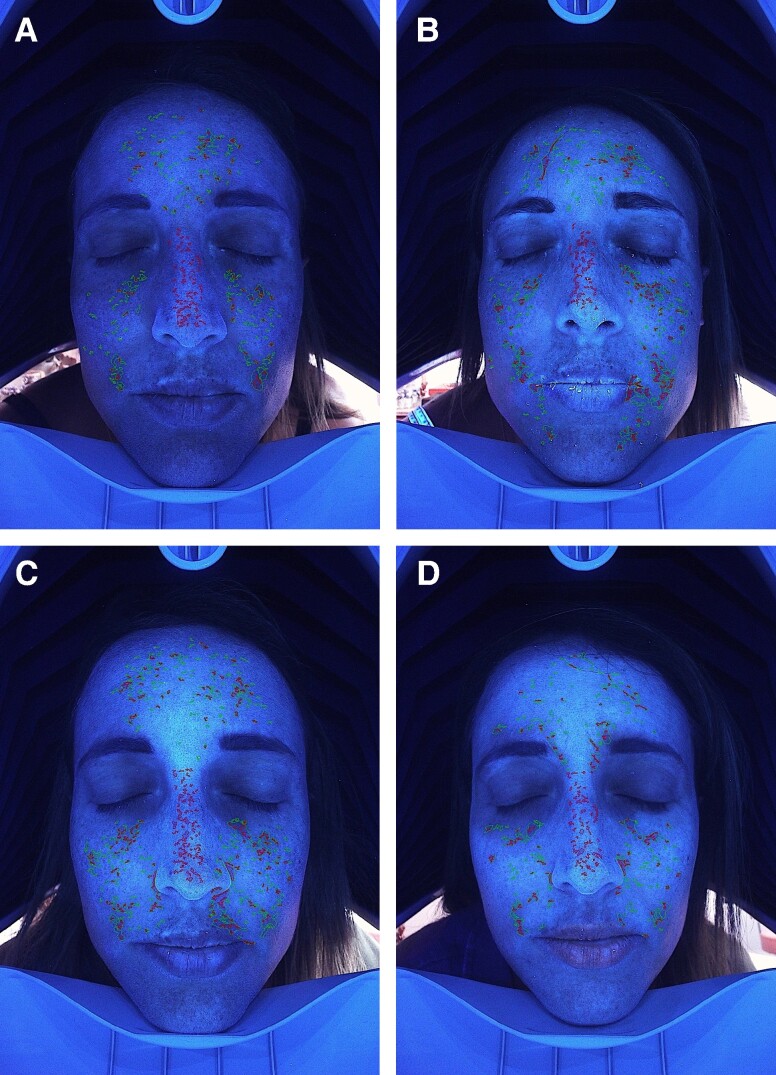
Imaging analysis of deep pigmentation, in a 44-year-old female patient. (A) The distribution of pigmentation, before the treatment. (B-D) The improvement of deep pigmentation at Weeks 3, 6, and 12, respectively.

**Figure 5.**
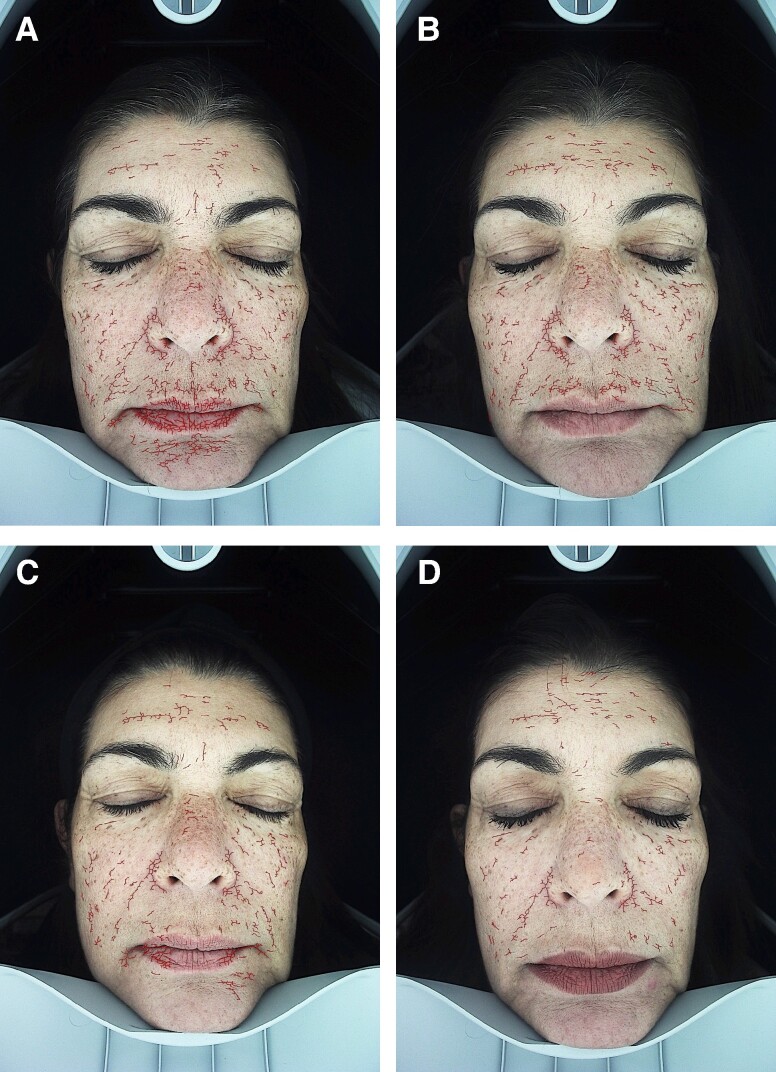
Imaging analysis of facial wrinkles, in a 57-year-old female patient. (A) The distribution of facial wrinkles before treatment. (B-D) The improvement of facial wrinkles at Weeks 3, 6, and 12, respectively.

**Figure 6.**
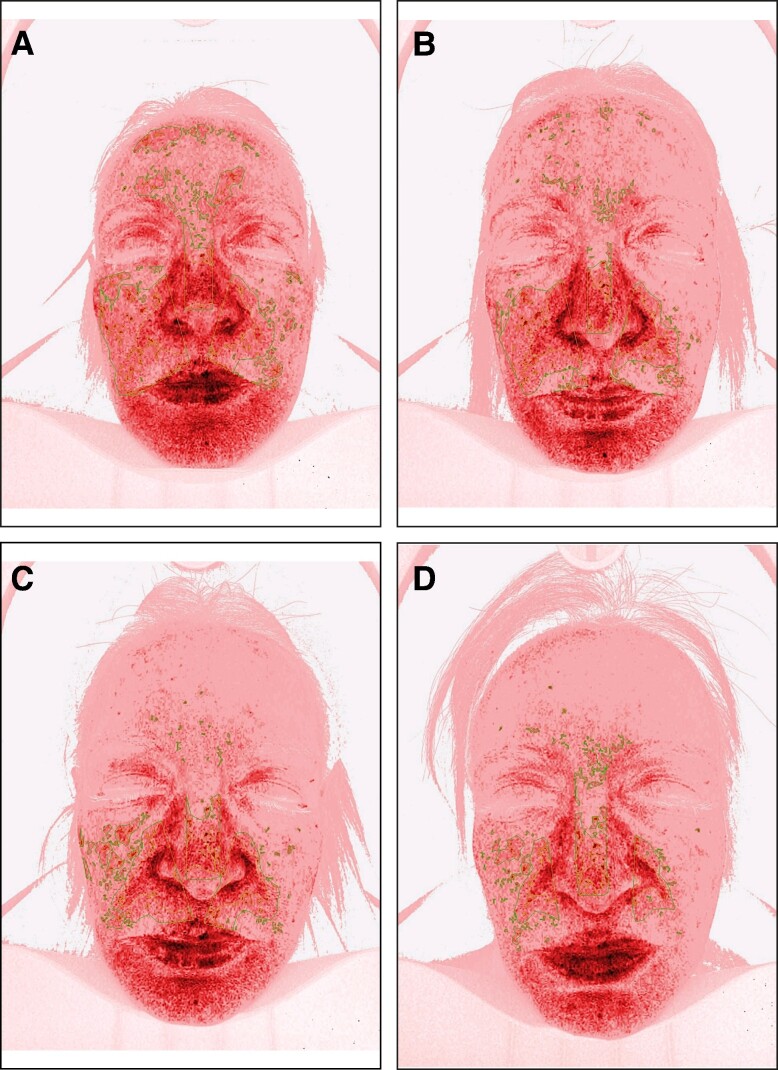
Imaging analysis of sensitive skin, in a 50-year-old female patient. (A) The intensity and distribution of sensitive skin, prone to rosacea, inflammation, and telangiectasia, before treatment. (B-D) The improvement of sensitive skin, at Weeks 3, 6, and 12, respectively.

When compared with baseline, imaging analysis showed significant improvement of facial pigmentation and signs of aging, by the end of study (Friedman's test, *x*^2^ = 35.441, *P* = .000; Figures 2-4). More precisely, surface skin pigmentation including solar lentigines and freckles significantly reduced in pigmentation intensity and skin surface area by Week 6 (*x*^2^ = 2.042, adj. *P* = .001), while this skin improvement continued up to Week 12 (*x*^2^ = 2.917, adj. *P* = .000). The total percentage of superficial dark spots significantly decreased at Week 12 (mean sp% at week_12_ = 26.8 ± 5.9), compared with baseline (mean sp% at baseline = 39.75 ± 8.9). Similar patterns of improvement were noted in deep-pigmented skin lesions, such as melasma and PIH (Friedman's test, *x*^2^ = 35.441, *P* = .000). Notably, the surface area and pigmentation intensity of deep-pigmented lesions significantly improved by Week 6 (*x*^2^ = 2.000, adj. *P* = .001), and this positive depigmentation effect of the treatment continued up to the end of the study, with no relapses (*x*^2^ = 2.917, adj. *P* = .000). The total dp% improved by 15.9% by the end of the study (mean dp% baseline = 45.33 ± 10.82, mean dp% week_12_ = 29.42 ± 7.19). Interestingly, compared with baseline, we observed marked improvement of red areas, which indicate inflammation, telangiectasia, and rosacea (Friedman's test, *x*^2^ = 35.415, *P* = .000). Even from the first treatment, there was a significant reduction in skin redness and inflammation (*x*^2^ = 1.917, adj. *P* = .002). There was a continuous improvement of the hyperactive vascular component of the skin across study visits (Week 6: *x*^2^ = 2.000, adj. *P* = .001; Week 12: *x*^2^ = 2.958, adj. *P* = .000) and an overall 7.34% reduction of sensitive, erythematous skin (mean sr% baseline = 47.17 ± 10.41, mean sr% week_12_ = 39.83 ± 10.36; Figures 2, 5). When we evaluated the antiwrinkle effects of the treatment, we saw a significant reduction of rhytides by the end of the study (Friedman's test, *x*^2^ = 31.800, *P* = .000). Depth and number of wrinkles markedly improved by week (*x*^2^ = −1.792, adj. *P* = .004; Figures 2, 6). By the end of the study, there was a notable improvement of skin quality by 6.3% (*x*^2^ = −2.542, adj. *P* = .000; mean nw% baseline = 52.3 ± 12.78, compared with mean nw% week_12_ = 58.67 ± 12.05).

### Psychometric Evaluation

**Figure 7.**
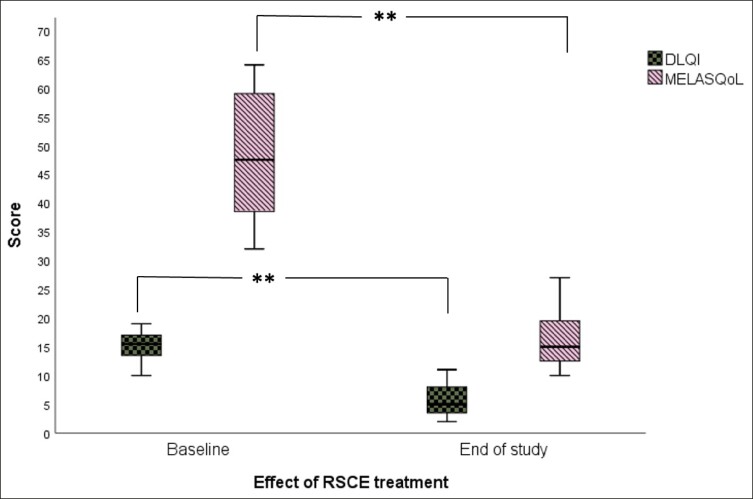
The effect of rose stem-cell-derived exosomes (RSCE) on the psychosocial functioning of patients with hyperpigmentation. The diagram shows the decrease in Dermatology Life Quality Index (DLQI) and Melasma Quality of Life scale (MELASQoL), at the end of the study (Week 12), indicating improvement of self-perceptions and psychosocial functioning posttreatment. Patients underwent 3 subsequent treatments at baseline, Week 3, and Week 6, with topical application of RSCE and microneedling. **P < .01.

According to our data, there was a marked improvement in total DLQI and MELASQoL scores at the end of the study, with 100% of patients reporting “more confident” and “socially more active” after treatment (Wilcoxon signed-rank test; DLQI: *Z* = −3.075, *P* = .002; MELASQoL: *Z* = −3.063; *P* = .002). Mean DLQI scores decreased by 10 points, indicating a significant improvement of HRQOL, from severe psychological impact to a “no to minor” effect on patients’ life (mean DLQI at week_12_ = 5.8 ± 2.8, mean DLQI at baseline = 15.2 ± 2.6; Figure 7). Similar patterns were observed at the MELASQoL scale, with a mean reduction of 30 points at the end of the study (mean MELASQoL at week_12_ = 16.2 ± 5.0, mean MELASQoL at baseline = 48.1 ± 10.7; Figure 7). In line with previous studies, MELASQoL scores were significantly correlated with DLQI scores (Spearman's coefficient = 0.945, *P* = .000).

## DISCUSSION

Uneven skin pigmentation of the face or facial hyperpigmentation has always been a common skin complaint. Patients with hyperpigmented lesions have an increased awareness about enhancing their skin quality and removing the dyspigmented skin.^26,27^

**Figure 8.**
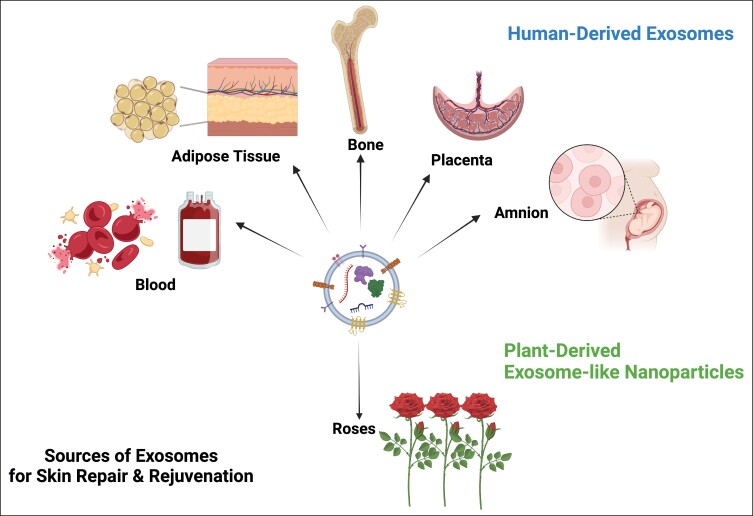
Sources of exosome-derived products currently used for skin rejuvenation. The figure shows the different sources (human and plant) of exosomes for the skin, currently used by healthcare providers in many different countries of the globe, while having relevant benchmark and/or clinical evidence.

On a cellular level, skin pigmentation is complex and regulated by multiple intracellular signaling systems, whereas exposure to UV light is the main cause of hyperpigmented skin. In response to the previous, there are numerous cosmetic compounds, in addition to facial aesthetic treatments which target pigmentation, yet with limitations in effectiveness and longevity. Increasing evidence from recent preclinical studies supports the potential of extracellular vesicles (EVs) mainly from human sources to improve skin pigmentation, by interfering with melanin production and promoting melanosome autophagy^10-13,28-32^ (Figure 8). In addition, these studies demonstrate the antiscarring, antiinflammatory, and antiaging effects of exosomes on in vitro and ex vivo preclinical skin models. Multiple plant-derived products exist as cosmeceutical and nutraceuticals for the skin, yet little is known for the plant-derived EVs. Recent evidence has shed light on their antiinflammatory, anticarcinogenic functions, as well as their immune modulating role in mammalian gut microbiota and regenerative, antiinflammatory, whitening properties on human skin.^33-35^ These findings instigated the interest in the use of plant EVs in aesthetics, highlighting their animal-free nature. On the contrary, to date, there are very few clinical studies on the effects of EVs on human skin and these are mainly on ASCE products,^10-12^ 1 pilot study on hPMSC-exos,^13^ 1 clinical study on platelet exosomes,^32^ and 1 sole study on plant-derived EVs from *Codium fragile* and *Sargassum fusiforme* seaweeds.^36^ To add to the previous, due to current worldwide restrictions on the use of EVs in clinical setting, these clinical studies were mostly performed on Asian skin, where regulatory bodies are allowing the use of both plant as well as mammalian source EVs in Medical Aesthetics. The current study, described in this paper, is the first to have demonstrated the positive effects of plant-derived EVs on Caucasian/Mediterranean skin. At the same time, it is the first clinical study using the combination of RSCE with microneedling to demonstrate efficacy on various clinical cases of pigmentation primarily on middle aged, photodamaged skin, and secondarily to show significant antiaging effects of this treatment on human skin, with a follow-up period of 6 weeks (Figures 1, 2).

**Figure 9.**
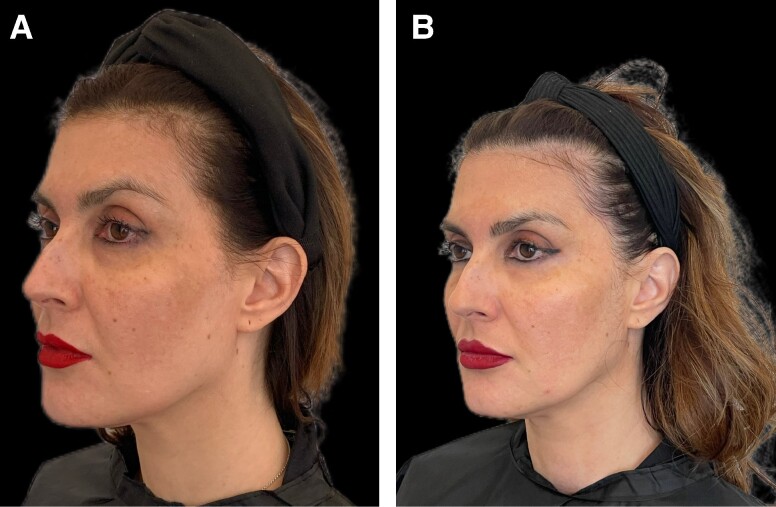
Clinical photography of a 49-year-old female patient, with melasma: (A) before treatment and (B) at the end of the study.

**Figure 10.**
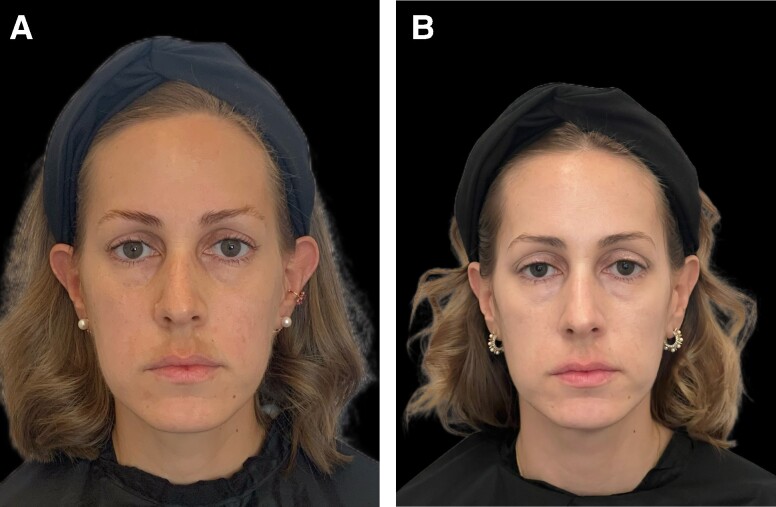
Clinical photography of a 39-year-old female patient, with postinflammatory hyperpigmentation after laser hair removal and melasma postpregnancy: (A) before treatment and (B) at the end of the study.

**Figure 11.**
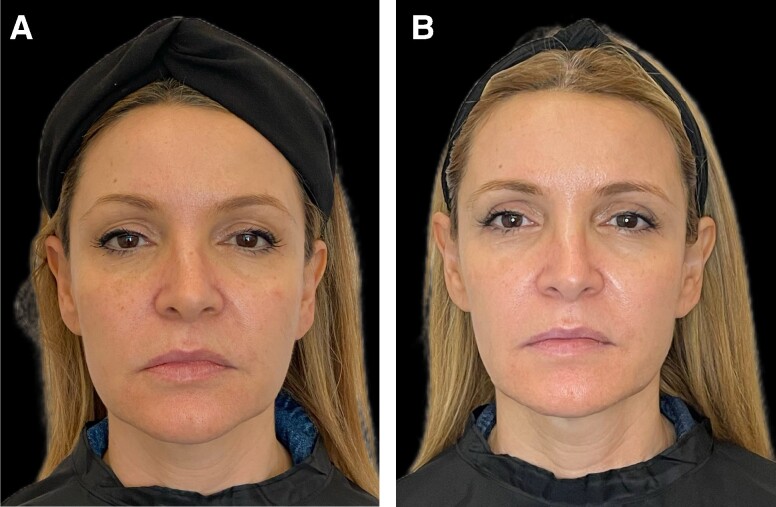
Clinical photography of a 50-year-old female patient with photodamaged skin: (A) before treatment and (B) at the end of the study.

Importantly, in our study, the source of exosomes is unique and should be elucidated here. Roses, in general, are symbols of love, beauty, inspiration, and purity, while righteously named the “Queens of flowers.”^37^ From head to pedals, roses have been extensively used in food industry, perfumery, and cosmetics. There are 4 species of roses, with *Rosa damascena* being the most important, not just for its intense scent, but also for its therapeutic properties. The phytochemical profile of *R. damascena* includes flavonoids, terpenes, carotenoids, tocopherols, glycosides, anthocyanins, quercetin, vitamin C, fatty oils, and other phenolic ingredients.^38^ There are multiple medicinal applications from the different formulations of *R. damascena* (rose oil, rose water, dried leaves, hips, and others), which are attributed to their phenolic ingredients, and have therefore been used as antioxidant, antitumor, antiinflammatory, analgesic, antimicrobial, and antiaging compounds.^39-41^ On the other hand, other studies suggest that *R. damascena* ethanolic extracts have a direct cytotoxic effects on human cells, and hence, the anticarcinogenic effects, but on the whole, they may be harmful for human skin cells.^38^ Gavra et al in vitro and clinical studies on different topical nanoparticle formulations of *Rosa* species, concluded that the lyophilized nanoparticle extract of *R. damascena* is the least cytotoxic to human keratinocytes, while the most effective clinically in their psoriasis cohort.^42^ Compared with placebo, psoriasis patients using this topical formulation had a significant improvement of the psoriasis area severity index and DLQI, after 6 weeks of treatment. To add to the previous, Won et al^14^ investigated in vitro, the EVs released by *R. damascena* callus supernatants and were able to identify the presence of exosomes that were nontoxic on human skin and hair cells, while increased the proliferation of human fibroblast resulting in an increased production of collagen. Notably, the authors provided evidence of the supernatant being toxic to human dermal papilla cells compared with the nontoxic exosomes released from the rose stem cells. When these RSCE were uptaken by human melanocytes, this resulted in a decreased production of melanin, suggesting an antipigmentary effect on skin. Bioinformatics analysis of the exosomal cargo showed many similarities between micro-RNAs (miRNA) with human, especially those of the Let-7 family, which is known for its antitumor properties. Other identified RSCE miRNAs were linked to antiinflammatory, cell proliferative and signaling human pathways (miR-8484, miR-574-5p, and miR-1246) that can fight cancer. Similar findings on the antiinflammatory role of RSCE were provided on the same paper. When RSCE were uptaken by macrophages, there was a significant reduction in the proinflammatory IL-6. Based on the previous data, our study is the first to elucidate the role of RSCE on human skin and confirm the abovementioned experimental results. We used a lyophilized compound of RSCE, as various studies have supported that lyophilization, compared with freezing, does not affect the EVs size and biomarker expression.^43^ The RSCE product used in the current study was a lyophilized compound of exosomes, isolated using the tangential flow filtration method (ExoSCRT), ensuring that the cellular debris and other unwanted particles of the conditioned media are not included in the end product. In fact, the whitening effect of RSCE on human skin was confirmed by our clinical results on various pigmentary conditions (PIH, melasma, sun-damaged skin; Figures 3, 4, 9-11). There was a significant improvement in the uneven skin tone in all patients, documented by both the patients and the independent evaluator, while confirmed by a multiple spectra imaging analysis system (Figure 1). The antiinflammatory effect of RSCE on human skin was observed on the imaging analysis, where results showed significant improvement of the sensitive, erythematous skin areas even after the first treatment (Figures 2, 5). At baseline, these sensitive, erythematous skin areas corresponded with visible hyperpigmented lesions, rosacea, as well as areas of skin thinning (moderate-to-severe wrinkling), which at the end of the study, demonstrated a significant reduction per surface area (Figure 2). Recent research links melasma with an increased expression of vascular markers. The presence of telangiectasia and erythema within melasma lesions is perhaps explained by the increased expression of the vascular endothelial growth factor (VEGF), a major angiogenic molecule of the skin. Kim et al^44^ presented an increase in the expression of VEGF from skin biopsies of melasma lesions, linked with increased angiogenesis and vascular diameter. Further added, Cohen et al^45^ were the first to show a significant association between IL-6 and VEGF, with IL-6 inducing the transcription of VEGF. Other studies have reported a positive association of IL-6 with VEGF which leads to abnormal angiogenesis, mostly related to various malignancies.^46^ Given the fact that UV radiation induces the release of several melanogenic factors from fibroblast, including IL-6, the angiogenic component of melasma may be a defective vascular network, which perhaps can be prevented by interrupting the IL-6 pathway. Genomic and in vitro studies on RSCE have shown a direct negative link between RSCE and the expression of IL-6 by macrophages, whereas our clinical data reported a significant improvement of erythema and hyperactive vasculature, even from the first treatment of RSCE with microneedling (Figures 2, 7). Our data may support the therapeutic role of RSCE in normalizing the defective vasculature of melasma and, therefore, promote a more prolonged corrective effect. These assumptions are yet to be elucidated. Finally, in our study, there were no major adverse events reported by our participants. All patients were satisfied with treatment (Figure 1), whereas most commonly reported adverse events were mild erythema and mild skin irritation (mainly edema and pruritus) which resolved within hours. These results underline the safety of our treatment protocol and are in line with the relevant in vitro studies.

Our study is the first to have demonstrated the positive effects of the topical application of RSCE on skin, when combined with microneedling. Most importantly, it has demonstrated a significant reduction of visible as well as dormant pigmentary lesions, while the brightening effect continued during the total of the follow-up period, without rebounds (Figures 2-4). In line with previous studies using ASCE and microneedling,^12^ our results demonstrated a significant improvement of both deep and superficial pigmented lesions. Interestingly, our results showed a significant difference in melanin distribution by the second treatment (Week 6), with a 13% reduction in visible spots and 16% reduction in deep, dermal pigmented spots. Similar results were demonstrated by Park et al,^12^ where a 10% reduction in skin pigmentation was noted on treated skin, showing comparable effects of RSCE with ASCE, when combined with microneedling. Microneedling was used in this study as a transdermal delivery system of exosomes. As the primary outcome of our study measure was the improvement of different kinds of pigmentary lesions, including melasma, the use of microneedling is generally safer that heat-based energy devices, where sometimes melasma can flare up.^47,48^ Multiple studies on microneedling suggest it is a safe and effective technique to allow penetration of small active molecules into the dermis, through the stratum corneum, with significant enhancements of skin tone, hydration, and thickness. Several authors have suggested that the maximum penetrance of active molecules postmicroneedling is at 5 min.^49^ Our study protocol is in line with the previous indications, where RSCE were gently massaged to the skin after 5 min of treatment. To increase RSCE absorption, participants were placed under red light with a thin-layered mask infused with RSCE. Red light therapy represents a noninvasive treatment targeting inflammation and wound healing. Recent research suggests that red light induces vasodilation and smooth muscle relaxation, which may imply a quicker absorption of topically applied compounds on irradiated skin.^50^

Lastly, the psychosocial consequences of stigmatization in skin disease and aesthetic treatments have long been recognized.^51^ The skin, being the largest organ of the body, plays a crucial role in interpersonal relationships, with patients showing visible lesions (face, hands, scalp) being more stigmatized and psychosocially isolated compared with those with lesions in nonvisible parts of the skin. This is the first study to examine the psychosocial properties of pigmentation on patients following an exosome-based treatment. We have included patient-reported outcome measures, such as DLQI and MELASQoL, to capture the effect of uneven skin tone on quality of life and interpersonal relations and how this may have changed at the end of the treatment. Our results show that dyspigmented skin is a major concern for patients, with both DLQI and MELASQoL scored high at baseline, with a mean DLQI of 15 and a mean MELASQoL of 48 (Figure 7). These scores were significantly improved at the end of the study, with a mean improvement of 10 points in the DLQI and 30 points in MELASQoL (Figure 7). Participants also reported that treatment “gave them more confidence,” “felt more in their skin,” without having to undergo major treatment downtime or limitations. Previous studies have shown a good correlation between these 2 psychometric questionnaires, which was also confirmed by our data (Spearman's coefficient = 0.945, *P* = .000).

In conclusion, this is the first clinical study on RSCE with microneedling for the improvement of pigmentation and skin photoaging. There are several limitations that need to be considered. The small number of patients (*n* = 12) and the lack of a control group with microneedling alone are limiting factors for the interpretation of our results. To add to the previous, the self-report outcome measures, including the psychometric questionnaires and GAIS, might be subjective to self-assessment bias, which could potentially emphasize the results in a more positive direction. Moreover, the patients were all Caucasian origin which may limit the extrapolation of our findings in other ethnic groups. Further added, participants underwent treatment all over the face, compared with other studies using a split-face model to determine efficacy. Our goal was to provide homogeneous results to our patients, while ensuring that these results came from a larger surface area compared with hemiface studies. At the same time, our intention was not only to research but also to respect the severely impacted psychological backgrounds of our participants, who were reluctant to undergo a hemiface treatment. Furthermore, our secondary objective was to test whether plant-derived exosomes are safe and effective for skin pigmentation and photoaging, when compared with recently published results on human-derived exosomes. Based on the current lack of human-derived exosomes for skin rejuvenation in Europe, our comparative group was missing and therefore we relied on available data existing in the literature. Another limitation applies on clinical photography, which, despite taken under the same conditions and timing of the day may be affected by the natural light of the room, as well as the luminosity of the skin posttreatment. We did not use any source of artificial light or filter effects in our medical images, whereas we relied on the natural skin appearance of our patients. However, it should be highlighted here that natural light can be a critical factor that might enhance the validitiy of the findings. In our attempt to minimize further bias, the independent evaluator was blinded, but at the same time, it still remains a limitation that we were not able to include a second blinded evaluator to maximize the validity of our results.

Moreover, in our limitations, our methodology protocol was strict to exclude any confounding parameters from environmental factors, such as diet and UV exposure, so that our results could only reply to the treatment protocol.

## CONCLUSIONS

Skin hyperpigmentation is common and appears to be more than skin deep, as it can be rather distressing for the affected patients. Pathomechanism is complex, whereas there are a variety of treatments available. As patient number increases across all phototypes, there is a need for effective treatments, with minimal side effects and high fulfillment of patients’ satisfaction. Stem-cell-derived exosomes for the improvement of skin quality and disorders of pigmentation is an active area of research. According to evidence gathered from the current study, RSCE when combined with nonthermal microneedling has the potential to improve a variety of hyperpigmented lesions, enhance skin quality, reduce inflammation, and ensure high satisfaction rates. Our results highlight the importance of plant, stem-cell-derived exosomes for the treatment of skin hyperpigmentation and warrant further research in the future.
